# Daily repetitive sensory stimulation of the paretic hand for the treatment of sensorimotor deficits in patients with subacute stroke: RESET, a randomized, sham-controlled trial

**DOI:** 10.1186/s12883-017-1006-z

**Published:** 2018-01-09

**Authors:** Jan C. Kattenstroth, Tobias Kalisch, Matthias Sczesny-Kaiser, Wolfgang Greulich, Martin Tegenthoff, Hubert R. Dinse

**Affiliations:** 1 Institute for Neuroinformatik, Neural Plasticity Lab, Ruhr-University of Bochum, Bochum, Germany; 2 Department of Neurology, University Hospital Bergmannsheil, Ruhr-University Bochum, Bochum, Germany; 3 HELIOS Hospital Hagen Ambrock, Hagen, Germany; 4 Department of Neuroinformatik, Neural Plasticity Lab, Ruhr-University of Bochum, Building NB3, 44780 Bochum, Germany

**Keywords:** Neurorehabilitation, Neuroplasticity, Sensorimotor, Stroke, Repetitive sensory stimulation

## Abstract

**Background:**

Repetitive sensory stimulation (RSS) adapts the timing of stimulation protocols used in cellular studies to induce synaptic plasticity. In healthy subjects, RSS leads to widespread sensorimotor cortical reorganization paralleled by improved sensorimotor behavior. Here, we investigated whether RSS reduces sensorimotor upper limb impairment in patients with subacute stroke more effectively than conventional therapy.

**Methods:**

A single-blinded sham-controlled clinical trial assessed the effectiveness of RSS in treating sensorimotor deficits of the upper limbs. Patients with subacute unilateral ischemic stroke were randomly assigned to receive standard therapy in combination with RSS or with sham RSS. Patients were masked to treatment allocation. RSS consisted of intermittent 20 Hz electrical stimulation applied on the affected hand for 45 min/day, 5 days per week, for 2 weeks, and was transmitted using custom-made stimulation-gloves with built-in electrodes contacting each fingertip separately. Before and after the intervention, we assessed light-touch and tactile discrimination, proprioception, dexterity, grip force, and subtasks of the Jebsen Taylor hand-function test for the non-affected and the affected hand. Data from these quantitative tests were combined into a total performance index serving as primary outcome measure. In addition, tolerability and side effects of RSS intervention were recorded.

**Results:**

Seventy one eligible patients were enrolled and randomly assigned to receive RSS treatment (*n* = 35) or sham RSS (*n* = 36). Data of 25 patients were not completed because they were transferred to another hospital, resulting in *n* = 23 for each group. Before treatment, sensorimotor performance between groups was balanced (*p* = 0.237). After 2 weeks of the intervention, patients in the group receiving standard therapy with RSS showed significantly better restored sensorimotor function than the control group (standardized mean difference 0.57; 95% CI -0.013–1.16; *p* = 0.027) RSS treatment was superior in all domains tested. Repetitive sensory stimulation was well tolerated and accepted, and no adverse events were observed.

**Conclusions:**

Rehabilitation including RSS enhanced sensorimotor recovery more effectively than standard therapy alone. Rehabilitation outcome between the effects of RSS and standard therapy was largest for sensory and motor improvement; however, the results for proprioception and everyday tasks were encouraging warranting further studies in more severe patients.

**Trial registration:**

The trial was retrospectively registered January 31, 2012 under DRKS00003515 (https://www.drks.de/drks_web/navigate.do;jsessionid=AEE2585CCB82A22A2B285470B37C47C8?navigationId=results).

## Background

Sensorimotor impairment resulting from cerebral dysfunction has substantial physical, psychological, and social implications. However, according to a 2014 Cochrane review, no high-quality evidence is available to support interventions currently used as part of routine practice [1]. Triggered by substantial progress in understanding the neuroplasticity mechanisms underlying learning and rehabilitation [2, 3], numerous alternative strategies have been suggested and tested, many of which showed a moderate quality of evidence such as constraint-induced movement therapy, robot-assisted therapy, mirror therapy, central and peripheral nerve stimulation, and virtual reality approaches [1, 3–6]. There is growing evidence that high doses of intervention are more beneficial than low doses. However, rehabilitation outcome is often limited [1, 7].

Recent work in healthy human subjects demonstrated that intensive training may not be necessary to induce behavioral improvement; however, it can be effectively acquired using a complementary approach in which plasticity processes are driven in response to exposure to repetitive sensory stimulation (RSS) [8, 9]. RSS is an approach that targets the cortical areas that represent the site of sensory stimulation to facilitate the development of neuroplastic processes. For example, a few hours of RSS in healthy participants has been demonstrated to alter cortical maps and cortical excitability representing the sites of the RSS stimulation [10, 11]. This becomes possible through the use of long-term potentiation-like sensory stimulation protocols [12, 13].

Despite different backgrounds and rationales for use, the concept of sensory stimulation protocols to induce neuroplasticity processes has attracted substantial interest and is currently being investigated and applied in many laboratories; however, different laboratories use different terms and different stimulation protocols [9, 13–19]. From our perspective, the rationale behind RSS is using the broad knowledge of brain plasticity to design specific sensory stimulation protocols in humans to induce synaptic plasticity to alter perception and behavior. The concept is to translate protocols that induce plasticity at the cellular level, such as long-term potentiation (LTP) and long-term depression (LTD), into sensory stimulation protocols [8, 20]. Central to using RSS is its ability to drive and facilitate neuroplasticity processes [2, 3], a property shared by central stimulation methods such as intracortical microstimulation, transcranial direct current stimulation, and transcranial magnetic stimulation [21–24].

In cellular research, high-frequency stimulation is used to induce LTP, whereas low-frequency stimulation evokes LTD [25, 26]. In the present study, we used a LTP-like protocol consisting of intermittent high-frequency tactile stimulation, which has been used before in healthy subjects to drive improvements in tactile perceptual abilities [12] parallel to cortical reorganization. To explain the changes evoked by RSS, this specific form of stimulation was suggested to evoke LTP-like plasticity processes in the cortical regions representing the stimulated skin sites [8, 27]. As a result, synaptic transmission is altered and cortical processing is remodeled, facilitating the reactivation of the cortical tissue that has preserved some functionality. The behavioral outcome of these processes is reflected in behavioral recovery. Evidence from studies in healthy subjects demonstrated far-reaching cortical remodeling including changes in cortical excitability, expansion of cortical representational areas, and enhanced functional connectivity between the somatosensory and motor cortex [10, 11, 28–30]. Accordingly, the background behind RSS as used in this study differs from electrical mesh-glove stimulation or electrical therapeutic stimulation, although these procedures have been reported to have a beneficial outcome on cortical excitability or muscular strength. In contrast to whole hand stimulation [31, 32], stimulating the tips of the fingers, which are the most densely innervated, allows a very specific targeting of somatosensory cortical representational areas. In fact, available imaging and EEG (electroencephalography) data from healthy participants provide supporting evidence that RSS selectively activates areas in somatosensory and motor areas representing the fingers and the hand [10, 11, 28, 33].

Various forms of electrical stimulation exist that are currently used in rehabilitation with mixed results [1, 34–38]. For each of these approaches, a wide range of stimulation parameters are in use, and the underlying mechanisms mediating beneficial effects remain largely to be clarified. FES (functional electrical stimulation) is applied to induce contraction of muscles to support motor action. On the other hand, so-called therapeutic electrical stimulation methods are applied to improve performance after the termination of stimulation, such as NMES (neuromuscular electrical stimulation), EMG (electromyography) -triggered electrical stimulation (EMG-ES), and TENS (transcutaneous electrical nerve stimulation). While TENS was introduced for pain treatment, effects observed after NMES and EMG-ES are assumed to be related to repetitive muscle contractions. Accordingly, as described above, the underlying principle and the aimed target of RSS differs from that of FES, NMES, or TENS.

The effects of RSS protocols have been extensively explored in both healthy young and elderly adults. When applied to the fingers, substantial improvements in the tactile, haptic, proprioceptive, and sensorimotor performance parallel to cortical reorganization were demonstrated [10, 12, 14, 15, 28, 39]. The effectiveness of this form of learning is assumed to arise from using stimulation protocols optimized to alter synaptic transmission and efficacy [8, 9]. While a number of studies used RSS or variants of this approach in patients with subacute or chronic stroke, RSS has yet to be implemented in routine clinical practice. So far, available studies indicate mixed effects in the treatment of upper limb impairment [16–18, 27, 39–43]. These mixed results of sensory stimulation come from the fact that sensory stimulation as method is poorly defined. Sensory stimulation approaches employ various forms of stimulation and testing parameters, as well as quite varying treatment times ranging from single applications to long-term treatment. This almost certainly causes large variability in outcome parameters. From our view, a significant advantage of repetitive stimulation is its passive nature, which does not require active subject participation, making the intervention substantially easier to implement and more acceptable to the individual.

Therefore, here we aimed to address whether in routine clinical practice, repetitive stimulation reduces sensorimotor deficits following stroke more effectively than conventional therapy. Based on our previous data about the effectiveness of RSS [10, 14, 15, 28, 39–41], we hypothesized that the standard therapy with RSS is superior to standard therapy with sham RSS. Because stroke can affect diverse aspects of sensorimotor abilities, we undertook a broad objective behavioral assessment evaluating the sensory, proprioceptive, sensorimotor, and motor functions. In addition, we aimed to find out tolerability, acceptance, and possible side-effects of the glove-applied RSS intervention.

## Methods

### Study design

This randomized single-blinded sham-controlled clinical trial was designed as a proof-of-concept study evaluating the effectiveness, safety, and compliance. We recruited 71 patients with subacute ischemic stroke with contralateral sensorimotor impairment from the HELIOS rehabilitation clinic in Hagen Ambrock, Germany. The study was done in accordance with the ethical principles from the Declaration of Helsinki. It was approved by the Ethics Committee of Ruhr-University of Bochum. All patients provided verbal and written informed consent before participating. The trial was retrospectively registered January 31, 2012 under DRKS00003515 (https://www.drks.de/drks_web/navigate.do;jsessionid=AEE2585CCB82A22A2B285470B37C47C8?navigationId=results). Recruitment had started June 15, 2010, lasting until December 31, 2012).

### Patients

The main inclusion criteria were age of 40–70 years, a diagnosis of unilateral subacute ischemic stroke, i.e., a left or right medial cerebral artery infarction with contralateral sensorimotor deficits of the upper limbs 3 to 4 weeks post-ictus. Also, patients should have low levels of spasticity, and stimulation perception thresholds of at least 20 mA. All patients were right-handed. Patients with mild transient ischemic stroke lasting fewer than 24 h, hemorrhagic stroke, and carotid artery dissection, history of cerebrovascular disease, wearing a pacemaker, aphasia, or cognitive impairment that prevented completion of the assessment were excluded. Patients were recruited with the help of physio- and occupational therapists at the rehabilitation clinic. The most relevant criteria patients did not meet were spasticity and paresis. Because of difficulties with patient enrollment, we widened the age criterion to patients aged 30 to 90 years.

### Randomization and masking

Patients were randomly assigned to either the target or control group using block randomization. The selection was performed using a computer-generated random list of numbers. Randomization sequence was accessible to assessors of the main outcome parameters only. No attempts were made to balance for gender or the side of the stroke. The patients and assessors of clinical tests (modified Rankin Scale, National Institutes of Health Stroke Scale, modified Barthel Index, Medical Research Council Scale, Frenchay Arm Test, and Wolf Motor Function Test) were masked to the treatment allocation. There was no masking for assessors of the main outcome parameters. Individual RSS intervention was conducted and monitored by therapists, who had received a detailed instruction in handling RSS procedures. Table 1 shows the baseline characteristics of the study patients.

**Table 1:** Demographic and baseline characteristics

	RSS group (n = 23)	Sham RSS group (*n* = 23)
Sex		
• Male	18 (78%)	16 (70%)
• Female	5 (22%)	7 (30%)
Age (years)	64 (34–86)	59 (43–89)
White ethnic origin	23	23
Lesion side (Rt./Lt.)	9/14	13/10
Handedness (R/L/A)	23//	23//
mRS	3.35 ± 0.71	3.26 ± 0.75
NIHSS	5.48 ± 3.22	5.17 ± 2.23
mBI	64.8 ± 34.59	66.5 ± 34.23
MRCA	3.73 ± 2.68	3.98 ± 1.49
FAT	3.48 ± 6.19	3.50 ± 5.99
WMTF	11.76 ± 16.43	11.56 ± 14.91

depicts demographic and baseline characteristics. Data are mean (±SD) or number (%). Abbreviations: Lesion side (Rt./Lt.) = right hemisphere/left hemisphere; Handedness (R/L/A) = right handed, left handed, ambidextrous; mRS = modified Rankin Scale; NIHSS = National Institutes of Health Stroke Scale; mBI = modified Barthel Index; MRCA = Medical Research Council Scale; FAT = Frenchay Arm Test; WMTF = Wolf Motor Function Test

### Procedures

Interventions were commenced 3.5 weeks (median) after stroke. Patients allocated to the control group received the same standard stroke rehabilitation as patients allocated to the intervention (RSS) group. Patients received RSS with standard rehabilitation therapy (physio- and ergotherapy) or sham RSS with standard therapy (control). In case of sham RSS, patients assumed a subthreshold treatment, although zero mA was applied. RSS treatment was applied independent of the schedule of the standard therapy. Total RSS-treatment time for both groups was 2 weeks (10 days).

### Interventions

RSS and sham RSS were applied for 45 min daily on the affected hand of the patients from both treatment groups. The stimulation sequence was the same as described previously [12] and consisted of 20-Hz bursts for 1.4 s with 5-s inter-train intervals, and a ramp/fall time of 0.3 s and 0.2 ms pulse width. The pulse trains were delivered with 2-channel stimulation devices. To account for innervation of the fingers, the stimulation for the predominantly median nerve-innervated fingers d1-d3 (the thumb, index, and middle finger) and the predominantly ulnar nerve-innervated fingers d4 and d5 (ring and little finger) was separately controlled and delivered. The pulses were transmitted by custom-made stimulation gloves that had built-in electrodes (1 × 4 cm) located on the first and third segment of each finger (cathode proximal). In the RSS group, the intensity of the stimulation was set individually at the highest values that the patient could easily tolerate for an extended period, but without reaching pain levels. For sham RSS, the same stimulation parameters and the same stimulation gloves were used, except for the stimulation intensity, which was set at zero mA.

The standard rehabilitation therapy consisted of individualized programs depending on the degree and nature of patients’ sensorimotor impairment. Occupational therapy was applied according to the concepts of Bobath, Affolter, and Perfetti. ADL training (activities of daily living) consisted of self-care tasks such as bathing and showering, dressing, self-feeding, food preparation, and personal hygiene. Cognitive therapy and activation training were offered to facilitate the recovery for independent living. Special and curative education included pottery-making to foster the skills and abilities of patients, and to prepare them for coping with confinement.

To characterize the upper extremity performance of the patients prior to the study, we used several clinical scales closely related to the performance of tasks in everyday life: the modified Rankin Scale (ranging 0 to 6, 0 no symptoms), National Institutes of Health Stroke Scale (ranging 0 to 42, 0 no symptoms), modified Barthel Index (ranging 0 to 100, 100 no symptoms), Medical Research Council Scale (ranging 0 to 5, 5 no symptoms), Frenchay Arm Test (ranging 0 to 5, 5 no symptoms), and 15 tasks of the Wolf Motor Function Test (ranging 0 to 15, 15 no symptoms). According to these scales, patients were characterized by slight to moderate to severe moderate disability. All patients had some sensory loss of various degrees.

### Objective assessment of sensorimotor behavior

To quantify the behavior objectively, the following tests were performed before and after the end of treatment for the affected and the non-affected limbs. The term non-affected is meant to indicate the limb contralateral to the site of stroke, which does not exclude potential changes in performance. Testing was performed in one session with breaks in between, total time varied between 60 and 90 min.

### Tactile performance

Touch thresholds were evaluated by probing the fingertips of the left and right index finger with von Frey filaments (Marstocknervtest, Marburg, Germany) [27, 44]. The test kit contained 16 different filaments calibrated to forces ranging from 0.25–294 mN in the logarithmic scale. We used a staircase procedure during which patients were required to close their eyes and report when they perceived an indentation of the skin on their fingertips. The applied forces, starting with a noticeable stimulus, were decreased in a stepwise manner until the subjects no longer perceived the stimulus (lower boundary) and then increased until the stimulus was perceived again (upper boundary). This procedure was repeated 5 times resulting in 10 values that were averaged to provide the touch threshold.

The grating orientation task (GOT) was tested in 2 alternative forced-choice paradigms [27, 45]. A set of nine custom-made hemispherical plastic domes with gratings cut into their surfaces, i.e., parallel ridges and grooves of equal widths for each dome, were applied to the tip of the index finger using a holder with a calibrated spring (150 mN) to enable constant application force. The width of the ridges and grooves (spatial frequency) varied from 0.5 to 9.5 mm. Each dome was presented 20 times. Immediately after touching the plastic domes, patients were asked to report the perceived orientation. The grating discrimination threshold was defined as the level at which 75% of the responses were correct and was determined by interpolating between the groove widths with 75% correct responses. The performance at this level was midway between chance and perfect performance.

### Nine-hole peg test (9-HPT)

To measure upper extremity fine motor performance (dexterity), we used the 9-HPT, a brief standardized quantitative test of the upper extremity function [46]. We measured the time needed separately for placing the pegs in and out of holes.

### Grip strength

Grip strength was measured 3 consecutive times for each hand with a Jamar hand dynamometer (Sammons Preston Inc., Bolingbrook, IL). Subjects were asked to stand up and hold the dynamometer with the arms parallel to the body [47]. The final results were the average across 3 trials.

### Jebsen-Taylor hand function test (JTHFT)

For the assessment of the functional hand motor skills, we used JTHFT [48]. Three of the 7 JTHFT subtests were performed: (1) picking up small objects and placing them in a can (SOP); (2) picking up small objects with a teaspoon and placing them in a can (FEED); and (3) stacking checkers (STACK). The performance was evaluated based on the time needed to complete each subtest.

### Joint position sense (JPS) assessment

The JPS assessment was conducted using the “Bochum Joint Position Sense Assessment” (BJPSA) as reported previously [39]. Patients were asked to compare lightweight polystyrene balls of different diameters held in the affected hand to a reference ball held in their non-affected hand, and to report, without visual information, if the tested ball located in the affected hand was larger, smaller, or equal in volume. In 3 consecutive subtests, the complete set of polystyrene balls (diameters: 3 cm, 5 cm, 6 cm, 7 cm, 8 cm, 10 cm, and 12 cm) were compared to a small reference (diameter 5 cm), mid-sized reference (diameter 7 cm), and large-sized reference (diameter 12 cm). The performance was assessed by calculating the number of errors (ERRnumb, a total of 21 decisions) and the weight of errors (ERRweight, calculated as the volume difference between the reference and test object). Previous studies showed that ERRweight and ERRnumb are independent parameters characterizing the joint position sense [39].

### Outcomes

The primary outcome was the sensorimotor performance (total performance index – TPI) as obtained by a combination of 10 different quantitative objective tests shown in Tables 3 and 4, which were performed before and after the end of treatment for both the affected and the non-affected limb: touch thresholds, acuity threshold (grating orientation task thresholds), dexterity using 9-HPT, grip strength, and proprioceptive functions according to JPS assessment and subtests of JTHFT: picking up small objects and placing them in a can (SOP), picking up small objects with a teaspoon and placing them in a can (FEED), and stacking checkers (STACK).

To compare and average performances across all tests and all subjects in both groups, we calculated the normalized performance indices (IP) [49]. This approach has been used by Engeneer et al. some years ago to combine performance data across different tasks that have different dimensions which cannot be averaged [50]. In addition, pooling the performance obtained for each single task into a single total performance index increases statistical power. IPs were calculated for each subject and each test. IP was calculated using the formula (*wp-ip*)/(*wp-bp*), where for a given test independent of a specific time point of measurement *wp* is the worst performance of all subjects, *ip* is the individual performance, and *bp* is the best performance of all subjects. IPs ranged between 1 and 0, where the best IP was 1, and the worst was 0. The total performance index (TPI) was calculated by averaging IP data across all 10 tests performed.

The secondary outcome measures were the performances in the 4 domains covering similar functional domains. For the “Sensory” domain, IP data from the touch threshold and 2-point discrimination tests were averaged. For the “Motor” domain, the IP data from the grip strength and the 9-HPT were averaged. For the “Proprioception” domain, IP data from the number and weight of errors of the JPS tests were averaged. For the “Everyday” domain, IP data from the 3 JTHFT subtests were averaged.

The tertiary outcomes were self-reported assessments of patients from both groups on adverse effects were administered after the completion of treatment including the questions: “was RSS pleasant or unpleasant?” and “what was the type of sensation evoked by RSS?” The user feedback obtained by a custom-made questionnaire included the ease of use, perceived sensations during RSS, positive and negative aspects of RSS, willing to continue using after release from the hospital, and willing to recommend to others.

Post-assessments were conducted within the week directly after completing the 10 days of treatments (mean 2.9 ± 1.4 days).

### Statistical analysis

Data were checked for normal distribution using Shapiro-Wilk test. Descriptive statistics were compiled. Normally distributed data were reported as the mean and standard deviation (SD). We used Student’s t-test to detect the differences between the 2 groups after the intervention. For statistical evaluation of the demographic and baseline characteristics, we used Student’s t- and Chi-square tests. Moreover, we computed effect sizes according to Cohen’s d, and confidence intervals. As early intervention with RSS following a stroke has not yet been tested, sample size and power calculation was therefore based on effect sizes in the range of 0.4 to 0.8 obtained in previous studies on sensory and dexterity performance [27], unpublished data, as well as data from elderly age-matched healthy participants [51], which using an alpha of < .05 and a power of 80% resulted in a sample size of 32 per group. To accommodate the possible drop outs, we selected a sample size of 70. All calculations were performed using Microsoft Excel 2010 and SPSS version 18.0 and higher.

## Results

### Participants

**Fig. 1:**
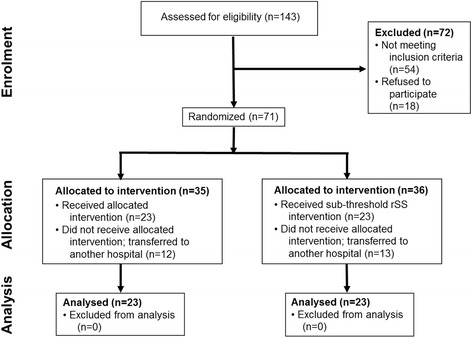
Consort flow diagram

We screened 143 patients with ischemic stroke for eligibility. Seventy-one of these patients were enrolled and randomly assigned to either the standard therapy with RSS (35 patients) or the control group receiving standard therapy with sham RSS (36 patients; see Fig. 1). Seventy-two were excluded (54 not meeting inclusion criteria, 18 refused to participate). In the RSS group, 23 of 35 patients completed the treatment; a similar proportion (23 of 36 patients) completed the treatment in the sham RSS group. Twelve (RSS group) and 13 patients (sham RSS group) did not complete their assigned treatment because they were transferred to another hospital or daycare. This transfer was solely caused by organizational reasons or personal reasons of the patients. The patients transferred showed no particularities and were within the range documented for the successfully treated patients according to the age, type of infarct, and severity of behavioral impairment. The data of 46 patients (*n* = 23 each group) were assessed for further statistical analysis. All patients received all interventions per protocol.

The baseline demographic and clinical characteristics were not statistically significantly different in both groups (Table 1). Both groups were balanced for age and gender. Most importantly, prior to the intervention, there were no differences in the clinical tests: the modified Rankin Scale, National Institutes of Health Stroke Scale, modified Barthel Index, Medical Research Council Scale, Frenchay Arm Test, and Wolf Motor Function Test. Furthermore, no significant differences were found in the averaged total performance at baseline.

### Stimulation-glove: Acceptance and side effects

The glove-based RSS treatment was well tolerated, and no negative side-effects were recorded. The average stimulation intensity was 10.4 ± 3.87 mA for the median nerve-innervated fingers, and 6.7 ± 3.46 mA for the ulnar nerve-innervated fingers. The sensations perceived during RSS were rated neutral to pleasant. The ease of use was reported as uncomplicated, possibly because in all cases, therapists assisted putting on the stimulation gloves. About two-third of the patients group were interested in continuing using RSS glove-treatment later in their homes.

### Average sensorimotor performance: Total performance score

As the first step in providing an overview of the effects of RSS versus sham RSS, we calculated the total performance index based on the performance of all 10 tests applied. An IP of 0 indicates the worst performance, characterized by the patient not being able to perform the task. An IP of 1 indicates the best performance observed for the non-affected limb. Accordingly, an IP of 0.5 indicates that the performance of the patient was at about 50%. The patients in both groups showed improvement; however, the beneficial effects were 2-fold higher in the RSS group compared to the sham RSS group (RSS: 22.9% gain from 0.56 to 0.68, *p* < 0.00005; sham RSS: 10.0% gain from 0.64 to 0.70, *p* < 0.05; the pre-post differences were significant between both groups using Student’s t-test; one-sided *p* = 0.027). While 17 out of 23 patients (74%) in the RSS group improved their performance by more than 0.05 points on the TPI scale, only 10 out of 23 patients (43%) in the sham RSS group showed such an improvement. Calculating the Standardized Mean Difference (SMD) revealed an effect size of 0.54 for RSS versus sham RSS therapy. The differential effectiveness was also apparent in the effect size calculated separately for each group, which was 0.55 for the RSS and 0.28 for the sham RSS groups.

To analyze how the positive effects were distributed across the more severe or, less severe patients, we subdivided the data according the median of the total performance index of both groups into more (TPI < 0.6; RSS *n* = 12; sham RSS *n* = 9) and less (TPI > 0.6; RSS *n* = 11; sham RSS *n* = 14) affected patients. For the RSS therapy, we found a pre-post improvement of 0.17 ± 0.04 (*p* = 0.001; 47.5% improvement) for the more severe patient subgroup and 0.07 ± 0.02 (*p* = 0.0005; 10.3% improvement) for the less severe patient subgroup. In contrast, for those receiving sham RSS, we found a pre-post improvement for the more severe patient subgroup of 0.08 ± 0.05 (*p* = 0.132; 23.6% improvement) and 0.04 ± 0.02 (*p* = 0.042; 5.6% improvement) for the less severe subgroup. These data indicate that the RSS therapy was particularly efficient in the more severe group of patients.

### Performance gain in the sensory and motor domains

Next, we analyzed how the effects of treatment were distributed across the four domains characterizing sensorimotor performance. Table 2 shows the pre- and post-IPs together with percent change and effect size for the 4 domains “sensory,” “motor,” “proprioception,” and “everyday tasks.” This breakdown of performance and performance gain into separate areas of sensorimotor performance showed that only the RSS therapy evoked significant improvement in all 4 domains indicative of a wide-range effectiveness targeting sensorimotor functions. In contrast, although the standard therapy resulted in measurable gains, only the changes in the performance of sensory and motor domains were significantly enhanced.

**Table 2:** Mean normalized performance index pre and post standard therapy with RSS and sham RSS

	Standard therapy with RSS					Standard therapy with sham RSS					Group difference
	pre	post	% gain	Cohens d	t-test	pre	post	% gain	Cohens d	t-test	t-test
Sensory	0.51 ± 0.24	0.68 ± 0.24	33.39	0.72	*p* = 0.0004	0.65 ± 0.23	0.70 ± 0.25	6.95	0.18	*p* = 0.034	*p* = 0.0044
Motor	0.50 ± 0.28	0.63 ± 0.24	27.26	0.52	*p* = 0.0001	0.54 ± 0.31	0.64 ± 0.28	17.42	0.31	*p* = 0.030	*p* = 0.087
Proprio	0.84 ± 0.12	0.88 ± 0.10	4.87	0.37	*p* = 0.022	0.87 ± 0.10	0.89 ± 0.07	2.40	0.25	*p* = 0.318	*p* = 0.220
everyday	0.50 ± 0.41	0.65 ± 0.36	30.63	0.40	*p* = 0.012	0.55 ± 0.39	0.68 ± 0.30	23.79	0.38	*p* = 0.194	*p* = 0.419

depicts mean (±SD) normalized performance index pre and post therapy effects of standard therapy with RSS (*N* = 23) and with sham RSS (N = 23) for the 4 domains tested. Mean (SD) normalized performance index pre and post therapy for the affected side are shown

The raw performance values for each of the 10 subtests performed are listed in Tables 3 ab and 4. Both treatment groups showed distinct improvement in the performance. However, while 9 out of 10 subtests in the RSS group showed significant improvement post-treatment, only 1 out of 10 subtests improved significantly in the standard treatment group.

**Table 3:** Raw data standard therapy with RSS vs. sham RSS – affected side

	standard therapy with RSS				standard therapy with sham RSS				
	PRE	POST	Cohens d	t-test pre-post	PRE	POST	Cohens d	t-test pre-post	t-test group difference
**a**									
Frey (mN)	87.73 ± 130.36	34.77 ± 85.75	0.48	0.035	48.91 ± 93.94	34.20 ± 83.72	0.17	0.318	0.083
GOT (mm)	7.50 ± 1.84	6.23 ± 2.26	0.61	<0.001	6.42 ± 2.31	5.95 ± 2.45	0.20	0.135	0.023
9-hole in (sec)	146.09 ± 90.24	108.83 ± 87.45	0.42	0.001	136.09 ± 95.38	95.87 ± 86.76	0.44	0.002	0.441
9-hole out (sec)	61.57 ± 85.69	27.65 ± 49.05	0.49	0.034	58.48 ± 85.92	44.00 ± 77.92	0.18	0.387	0.195
Grip strength (kg)	15.55 ± 10.77	20.18 ± 12.26	0.40	0.001	20.44 ± 13.56	21.28 ± 10.90	0.07	0.601	0.026
JTHFT Picking objects (sec)	65.24 ± 48.38	47.72 ± 44.93	0.38	0.034	54.20 ± 40.64	33.60 ± 28.78	0.58	0.106	0.386
JTHFT Feeding (sec)	73.91 ± 48.26	52.40 ± 47.50	0.45	0.031	68.55 ± 46.91	56.25 ± 49.71	0.25	0.327	0.286
JTHFT Stacking (sec)	51.03 ± 52.78	38.10 ± 49.72	0.25	0.295	40.069 ± 52.23	31.23 ± 48.19	0.19	0.500	0.442
**b**									
Frey (mN)	1.67 ± 1.95	1.66 ± 3.12	0.01	0.979	1.66 ± 4.38	1.46 ± 2.60	0.06	0.604	0.645
GOT (mm)	5.15 ± 2.53	4.61 ± 2.57	0.21	0.135	4.14 ± 2.07	3.98 ± 2.06	0.08	0.273	0.543
9-hole in (sec)	23.14 ± 6.95	20.23 ± 4.84	0.49	0.01	21.43 ± 8.72	20.43 ± 7.78	0.12	0.159	0.232
9-hole out (sec)	9.0 ± 2.45	8.14 ± 1.83	0.40	0.062	8.30 ± 2.87	8.22 ± 2.45	0.03	0.833	0.547
Grip strength (kg)	32.90 ± 8.63	34.75 ± 9.47	0.20	0.105	35.21 ± 11.43	34.60 ± 10.58	0.06	0.494	0.481
JTHFT Picking objects (sec)	12.60 ± 4.03	12.74 ± 3.52	0.04	0.973	12.12 ± 5.37	11.99 ± 4.56	0.03	0.759	0.373
JTHFT Feeding (sec)	16.08 ± 6.25	14.66 ± 4.51	0.26	0.269	16.09 ± 10.07	14.38 ± 6.84	0.20	0.172	0.649
JTHFT Stacking (sec)	4.07 ± 1.23	3.67 ± 1.22	0.32	0.298	3.64 ± 1.47	3.35 ± 1.01	0.23	0.136	0.354

*JHTFT* Jepsen Taylor hand function test

a and b depict the effects of standard therapy with RSS and standard therapy with sham RSS for all 8 unilateral tests used (N = 23 each). Mean (±SD) raw performance values (dimensions indicated in the left columns) pre- and post-therapy for the affected (a) and not affected (b) arm are shown

**Table 4:** Raw data standard therapy with RSS vs. sham RSS – bilateral task JPS = Joint Position Sense

standard therapy with RSS					standard therapy with sham RSS				
	PRE	POST	Cohens d	t-test pre-post	PRE	POST	Cohens d	t-test pre-post	t-test group difference
JPS Number of Errors	4.53 ± 4.52	3.90 ± 2.46	0.34	0.072	4.00 ± 2.67	3.53 ± 2.03	0.22	0.509	0.304
JPS Weight of Errors	205.95 ± 223.91	119.60 ± 210.35	0.37	0.0075	148.52 ± 152.34	108.90 ± 123.46	0.27	0.204	0.136

depicts the effects of standard therapy with RSS and standard therapy with sham RSS for the bilateral task testing the Joint Position Sense (N = 23 each). Mean (±SD) raw performance values (dimensions indicated in the left columns) pre- and post-therapy are shown

## Discussion

We demonstrated that the combined treatment of sensorimotor deficits in patients with subacute stroke using RSS in combination with standard therapy was superior to standard therapy with sham RSS. We used a broad range of quantitative assessments of sensorimotor performance to investigate the therapeutic potential of RSS, consisting of intermittent high-frequency tactile stimulation in combination with standard rehabilitation therapy. An advantage of RSS was observed in all domains tested evaluating sensory, motor, and proprioceptive as well as everyday task performance. Although sham RSS had positive effects in all 10 assessments used, only 1 in 10 showed significant improvement, while in the RSS group 9 of 10 tests showed significant improvement. The superior efficiency of the RSS therapy was mirrored in the effect sizes for the RSS and sham RSS (Cohen’s d). Although overall less patients could be analyzed than originally planned, we still could obtain significant differences between treatment groups. This was possible because of pooling the performance obtained for each single task into a single total performance index, which increased statistical power.

### RSS treatment using finger-tip stimulation gloves

For treatment, patients were equipped with custom-made stimulation gloves that had built-in electrodes contacting the first and third segment of each finger, enabling clearly defined stimulation of the fingertips. Accordingly, the gloves used employ a profoundly different strategy than the so-called mesh gloves or whole hand stimulation approaches, where the entire hand is diffusely stimulated. As stimulation thresholds vary between the median and ulnaris nerves innervating the fingers, a 2-channel stimulation device allowed for the separate adjustment of the stimulation intensities. We did not observe adverse events. All patients tolerated the gloves without problems. The evoked sensations on the fingers and hand were described as tickling or massaging. To exclude the possibility that offering a new therapy might bias patients [52], we used a sham protocol to control for the effects of RSS. This procedure allowed the use of the stimulation gloves in the control group without actual stimulation. Compared to a waiting group without employing the stimulation glove, our procedure can be regarded as more conservative in that it most likely preserves possible placebo effects mediated by using a new stimulation device.

### RSS effects on sensory and motor function

The largest gains were observed for sensory functions, which was not unexpected when using a sensory stimulation protocol. On the other hand, the improvement in the sensory functions was rather limited in the control group receiving standard therapy (33.3% vs. 6.9%). This latter finding fits with previous studies that reported that following motor rehabilitation, beneficial effects on pure sensory functions are small. Given that learning and re-learning skilled motor behavior requires largely intact somatosensory input processing, it is conceivable that when the sensation is corrupted, motor recovery is corrupted as well. It has, therefore, been assumed that patients with somatosensory deficits following stroke experience more persistent motor impairment than those without such deficits [5].

The RSS group improved not only sensory functions to a greater extent than the sham RSS group alone but also motor recovery, which was similarly better in the RSS group (27.2% vs. 17.4%). The effectiveness of RSS in improving motor function was also demonstrated for healthy adults and elderly individuals [51, 53, 54]. However, how RSS affects the motor system requires further research. It is assumed, for example, that the transfer of beneficial effects to sensorimotor behavior is based on the interconnections between the somatosensory and motor cortex [55, 56], which in turn elicits reorganization in the motor cortex. Moreover, dexterity tasks, such as the pegboard task, require intact tactile inputs for fine manipulation. In such cases, the net motor behavior is dependent on working tactile functions. We, therefore, assume that a combination of task-inherent tactile-motor requirements together with motor cortex reorganization results in the motor recovery observed following RSS.

In the present study, no follow-up assessment was planned; however, outcome measures were obtained within the week following the 10 days of treatment. However, other studies employing RSS treatment have shown that in patients with chronic stroke, restoration effects were fully preserved in a 4-week follow-up [41].

It should be noted that comparable beneficial effects have been reported following RSS applied alone, without any parallel standard therapy. In patients with chronic stroke, improvement in the sensory and motor abilities was reported following 4 weeks of RSS treatment [41]. In patients with chronic brain injury where the cerebrovascular dysfunctions dated as far back as 13 years, the application of RSS for up to 76 weeks showed substantial improvements in the sensory and motor abilities [27].

### Comparison with other therapeutic measures

Most rehabilitation strategies for upper extremity stroke patients target motor recovery. However, among the many sources that contribute to incomplete recovery, poor recovery of somatosensation might play a crucial role. There is agreement that intact afferent sensory information is not only crucial for tactile and haptic but also for motor performance [7, 57]. As a consequence, maintained compromised sensory abilities further complicate motor function recovery. According to a recent systematic review on upper extremity motor recovery, more than 10 different forms of interventions had been reviewed including fitness training or physical fitness training, repetitive task training, electrostimulation, biofeedback, force and position feedback, bilateral training, constraint-induced movement therapy, or mental practice with motor imagery [5]. SMDs for a 95% CI differed widely ranging from 0.09 for upper limb high intensity training to 0.84 for mental practicing with motor imagery, or 0.47 for electrostimulation (arm function) and 0.12 hand function (cf. 0.57 as found in our present study). A similar difference for hand and arm function was found following constraint-induced movement therapy (0.17 vs. 0.73). As the authors concluded, these data indicate that large amount of research is still required to define much more clearly the interventions that carry most benefits.

### Implication for everyday life

An issue of substantial relevance is the question in how far improvements that can be measured in a laboratory or clinical surrounding are transferable into everyday life, which constitutes the major challenge of all intervention strategies. In a recent case study with patients with chronic brain injuries, comparable effect sizes for gains of sensorimotor performance as reported here were obtained using RSS treatment [27]. One patient reported subjectively improved sensations when touching object surfaces with the fingers, which had not been possible before the intervention. In contrast, another patient showed improvements in all investigated aspects of sensorimotor performance, but reported little impact on changes of everyday life activity of the affected extremity. It is conceivable that this patient got used to not involving the affected hand and arm, a phenomenon captured in the concept of “compensatory learned non-use of the affected limb” [58, 59]. As a consequence, such individuals might not be able to recognize gains of sensorimotor performance induced by an intervention although they can be demonstrated under laboratory conditions. Given that, further investigations are necessary to improve therapeutic programs to facilitate re-using affected limbs.

### Comparing affected and non-affected side

A detailed analysis of the baseline performance and treatment effects of the non-affected side were beyond the scope of this study. However, after the 2 weeks of treatment, patients of both groups improved their performance to a variable degree. Further studies are needed to investigate in how far this improvement is due to mere task learning and practicing. Alternatively, it is conceivable that neural reorganization of the stroke-affected cortical sites also mediated behavioral changes of the contralateral limb.

### Strengths and limitations

Overall this study is limited by the substantial number of patients that were transferred to other hospitals, which thus could not participate in the study. After 2 weeks of the intervention, patients in the group receiving standard therapy with RSS showed better restored sensorimotor function than the control group as indicated by significant differences in the total performance index. However, when comparing group differences for the data obtained for each of the ten different tasks, only 2 tasks (2-point discrimination and grip strength) were significantly different, although the within group pre-post comparisons indicated much stronger benefits for the RSS therapy. This discrepancy is most likely due to the huge interindividual variability. It is conceivable that having included the additional patients that had been transferred to other locations, the resulting higher statistical power would have revealed significant differences for the other tasks as well. Despite the fact that both groups were statistically balanced, the patients in the standard therapy group with sham RSS were slightly less affected. It is therefore possible that the degree of affectedness might impose some influence on the overall outcome of this study. However, the recorded beneficial effects of the RSS group will inform future trials. The main strength of this study is that we assessed a broad range of parameters characterizing sensorimotor behavior objectively, and reported the effects of RSS and sham RSS not only separately for these markers, but that we combined them into domains characterizing diverse aspects of sensorimotor behavior. Another limitation is that we did not include patients with more severe impairments. This, together with larger samples sizes to accommodate the high interindividual variability present in this group of subacute stroke patients, is recommended in future trials.

## Conclusion

This randomized sham-controlled clinical study demonstrated that application of RSS using an LTP-like protocol in combination with standard therapy enhances a broad range of sensorimotor performances of the upper limb in patients with subacute stroke over a treatment period of only 2 weeks. Furthermore, this outcome was superior to the effects observed with the standard therapy alone. Patients tolerated the RSS treatment well, as well as the RSS application using a custom-made stimulation glove. Further studies are needed to obtain more detailed insight into the mechanisms underlying this approach. In addition, future trials will have to explore effectiveness in more severe patients, and additional beneficial effects might be obtained when using RSS as a priming procedure before behavioral training. Independent of this, the present data, when confirmed by future studies, suggest that the application of RSS combined with standard therapy might be another promising approach in the treatment of sensorimotor impairments after stroke compared to the current standard treatment practice.

## Abbreviations

9-HPT: Nine-Hole Peg Test
ADL: Activities of daily living
BJPSA: Bochum Joint Position Sense Assessment
CI: Confidence intervals
d1: Thumb
d2: Index finger
d3: Middle finger
d4: Ring finger
d5: Little finger
EEG: Electroencephalography
EMG: Electromyography
ERRnumb: Number of errors (Bochum Joint Position Sense Assessment)
ERRweight: Weight of errors (Bochum Joint Position Sense Assessment)
FAT: Frenchay Arm Test
FEED: Picking up small objects with a teaspoon (Jebsen-Taylor Hand Function Test)
FES: Functional electrical stimulation
GOT: Grating orientation task
IP: Normalized performance indices
JPS: Joint position sense
JTHFT: Jebsen-Taylor Hand Function Test
LTD: Long-term depression
LTP: Long-term potentiation
mBI: Modified Barthel Index
MRCA: Medical Research Council Scale
mRS: Modified Rankin Scale
NIHSS: National Institutes of Health Stroke Scale
NMES: Neuromuscular electrical stimulation
R/L/A: Right handed/left handed/ambidextrous
RSS: Repetitive sensory stimulation
SD: Standard deviation
SMD: Standardized mean difference
SOP: Picking up small objects (Jebsen-Taylor Hand Function Test)
STACK: Stacking checkers (Jebsen-Taylor Hand Function Test)
TENS: Transcutaneous electrical nerve stimulation
TPI: Total performance index
WMFT: Wolf Motor Function Test
